# Advantages and artifacts of high-speed OLED monitors for vision, eye-tracking, and EEG research

**DOI:** 10.3758/s13428-026-03034-9

**Published:** 2026-05-11

**Authors:** Olaf Dimigen, Arne Stein

**Affiliations:** 1 Department of Experimental Psychology, University of Groningen, Grote Kruisstraat 2/1, 9712 TS Groningen, The Netherlands; 2 Research School of Behavioural and Cognitive Neurosciences, Faculty of Science and Engineering, University of Groningen, Groningen, The Netherlands

**Keywords:** Laboratory monitor, Organic light-emitting diode (OLED), Visual psychophysics, Vision science, Gaze-contingent display updates, OLED temperature artifacts

## Abstract

The recent introduction of organic light-emitting diode (OLED) monitors with refresh rates of 240 Hz or more opens new possibilities for their use as precise stimulation devices in vision research, experimental psychology, and electrophysiology. These affordable high-speed monitors, targeted at video gamers, promise several advantages over cathode ray tube (CRT) and liquid crystal display (LCD) monitors. Unlike LCDs, OLEDs have self-emitting pixels that can show true black, resulting in superior contrast, a broad color spectrum, and wide viewing angles. More importantly, the latest OLEDs offer excellent timing properties with minimal input lag and rapid transition times. However, OLED technology also has potential drawbacks, such as auto-brightness limiting (ABL), where luminance changes with the number of illuminated pixels. This study characterized a 240 Hz OLED monitor (ASUS PG27AQDM) in terms of its timing, temporal independence, spatial uniformity, viewing angles, warm-up time, and ABL behavior, and compared it with CRTs and LCDs. Results confirm excellent temporal performance, with CRT-like transition times, wide viewing angles, and good spatial uniformity. We show that ABL can be prevented with appropriate settings. However, we also report a novel type of luminance artifact on OLEDs, where high-contrast stimuli, shown for long durations, can create image persistence via localized warming or cooling of the panel. Finally, we demonstrate the monitor’s benefits in two time-critical paradigms: rapid invisible flicker tagging (RIFT) and saccade-contingent display changes. The latest consumer OLEDs provide precise and cost-effective stimulation devices for time-critical experiments, although some caution is warranted in experiments involving long exposures to high-contrast stimuli.

## Introduction

Research in visual perception and experimental psychology requires displays with short onset latencies and accurate timing. Fast and consistent stimulation is also critical for measuring psychophysiological responses, such as those in electroencephalography (EEG). Due to their excellent temporal properties, cathode ray tube monitors (CRTs) have long served as the gold standard in these fields, but CRTs in good working conditions are increasingly hard to find. Also, while their pulsed emissions provide exact stimulus onsets, CRTs do not support the display of truly continuous luminance patterns (Elze, 2010b). They additionally suffer from poor spatial independence (e.g., Pelli, 1997), and their phosphor persistence can delay stimulus offsets (Brainard et al., 2002). In many laboratories, CRTs have now been replaced by liquid crystal displays (LCDs), which come with their own drawbacks. In LCDs, each subpixel contains liquid crystals that twist or untwist to pass through or block the light emitted by a backlight (Elze & Tanner, 2012). Because this reorientation of the liquid crystals takes time, LCDs have comparatively sluggish response times that depend on the content displayed during the previous frame (poor temporal independence; Elze & Tanner, 2012; Hallum & Cloherty, 2021). The use of a backlight also means that LCDs cannot display true black, limiting their contrast. Furthermore, LCDs typically show a strong drop-off in luminance at wide viewing angles (e.g., Ghodrati et al., 2015; Zhang et al., 2018). Since these issues are inherent to LCD technology, they also affect modern gaming LCDs (Zhang et al., 2018) as well as specialized LCDs designed for vision research or EEG (Ghodrati et al., 2015).

### Potential advantages of organic light-emitting diode (OLED) monitors

An alternative display technology increasingly used in consumer products is the organic light-emitting diode panel, simply called “OLED” in the following. OLEDs use organic compounds that directly emit light when subjected to an electric current, which allows for precise control over each (sub)pixel's output. The absence of a backlight also means that pixels can show true black, supporting practically infinite contrast ratios, large viewing angles, and a wide color spectrum. Most importantly, OLEDs promise excellent timing properties (Elze et al., 2013), with fast and temporally independent transition times.

The potential of OLEDs as stimulation devices for visual research was already demonstrated more than a decade ago (Cooper et al., 2013; Elze et al., 2013; Ito et al., 2013; Matsumoto et al., 2014). However, the panels characterized in these early studies were still technically limited in many ways—in particular, by supporting effective refresh rates of only 60 Hz. This limits their usefulness in paradigms requiring fine gradations in stimulus duration (Poth et al., 2018) such as visual masking, rapid serial visual presentation, multisensory integration, temporary order judgments, or other assessments of psychophysical thresholds.

In late 2023, a new generation of high-speed OLEDs entered the market. Aimed at video gamers, these affordable consumer-grade monitors offer refresh rates of 240 Hz or even 480 Hz (Dimigen et al., 2026) and are advertised as having extremely short input lags (the latency from sending the graphics processing unit [GPU] command to the onset of the monitor’s response) and fast transition times (the time from the start of the response until the desired color or gray level is reached). Together with the high image quality generally provided by OLED technology, this makes them interesting candidates for time-critical experiments on vision and cognition.

### Potential drawbacks of OLEDs for visual science

OLED panels also have potential drawbacks. For example, their organic compounds degrade faster than the inorganic materials used in CRTs or LCDs (Kam et al., 2023; Laaperi, 2008). In particular, screen regions where the same pattern persists for extended periods (e.g., taskbars, logos) face the risk of image retention from burn-in (Kam et al., 2023). Modern OLEDs include various functions to mitigate burn-in risk (see Baker, 2022, for an overview). These include the occasional shifting of the entire screen image by a few pixels (“pixel shifting”), the dimming of static screen regions, or so-called pixel cleaning cycles, during which pixels are rapidly turned on and off to remove residual charges. Because some of these features need to be deactivated for precise experimentation, it is unclear whether burn-in limits the monitor’s lifetime under laboratory conditions.

A second and more serious drawback of OLEDs are luminance saturation effects and spatial dependencies caused by the panel’s auto-brightness limiting (ABL) mechanism (Baker, 2022). To manage power consumption and thermal output, OLEDs dynamically adjust the luminance of presented stimuli according to the current average picture level (APL), the overall luminance output of all pixels (Baker, 2022). In other words, while the panel can display a small bright stimulus at maximum luminance, this becomes impossible with a large bright stimulus. ABL is an inherent technical limitation of OLED panels and not specific to the model tested here. However, different monitors will vary with regard to the APL at which this mechanism kicks in. With some OLEDs, ABL behavior can even be experienced during everyday computer use where the display will dim noticeably whenever a bright graphical user interface window is maximized to fill the screen.

Obviously, any ABL behavior is highly problematic for precise experimentation. In fact, ABL-like luminance saturation effects were already identified as a major drawback in earlier tests of OLED monitors for vision research and medical diagnostics (Cooper et al., 2013; Elze et al., 2013; Ito et al., 2013). However, considerable technical progress has been achieved with consumer-grade OLEDs since then. In particular, because current models support comparatively higher luminance levels, it should be possible to prevent ABL by operating them well below their peak brightness output (see also Cooper et al., 2013), where these monitors should still offer high contrasts.

### Current study

The goal of the present work was to evaluate the suitability of modern high-speed OLEDs for experimental research, with an emphasis on fast and time-critical paradigms. For this, we characterized one promising model, the ASUS ROG Swift OLED PG27AQDM, with a focus on its temporal properties, which we also compared to those of some CRT and LCD monitors available in our laboratory. In addition to conducting standard tests, we were especially interested in whether luminance artifacts due to ABL could be prevented. Finally, we assessed the monitor’s timing performance in two exemplary time-critical paradigms, detailed in the following.

### Practical test 1: Fast flicker

As a first practical test, we assessed the OLED monitor’s ability to faithfully reproduce fast flickering stimuli, where each luminance step is only shown for a single frame (4.17 ms). In electrophysiological research, flickering stimuli are commonly used to track visuospatial attention (Lalor et al., 2007; Morgan et al., 1996; Regan, 1966). A recent variant of this approach, rapid invisible frequency tagging (RIFT), measures the coherence between a sinusoidal, high-frequency (50–86 Hz) contrast- or luminance-modulated flicker sequence and neural responses in the magnetoencephalogram (Minarik et al., 2023; Seijdel et al., 2023) or EEG (Arora et al., 2024; Dimigen et al., 2026). A key advantage of RIFT is that the flicker remains barely visible (Minarik et al., 2023; Seijdel et al., 2023) due to the fast flicker rate and the more gradual contrast changes provided by the inclusion of intermediate gray levels within each flicker cycle. In studies to date, RIFT stimulation has necessitated the use of an expensive high-speed projector refreshing at up to 1,440 Hz (Minarik et al., 2023). Given their fast transition times, we tested whether a high-speed OLED can also faithfully present fast flicker sequences, in which the stimulus changes on every frame.

### Practical test 2: Saccade-contingent stimulation

Another type of studies that require low-latency stimulation are gaze-contingent eye-tracking paradigms in which the screen updates in real time with gaze position. For example, in one long-standing line of research, viewers see a preview of an object in parafoveal or peripheral vision (e.g., a word, a face, or a pattern of dots) before they make a saccadic eye movement towards it (McConkie & Rayner, 1975; McLaughlin, 1967; O’Regan & Lévy-Schoen, 1983; Rayner, 1975). To investigate the impact of the pre-saccadic preview on the following post-saccadic processing of the object at the center of gaze, these studies typically include an invalid preview condition where the object is exchanged, altered, or displaced during the saccade (Cox et al., 2005; e.g., Deubel et al., 1986; Herwig & Schneider, 2014; McConkie & Rayner, 1975; Rayner, 1975; C. Wolf & Schütz, 2015).

A major technical challenge with saccade-contingent paradigms is in timing the display change so that it happens during the eye movement, while the viewer’s visual thresholds are elevated. This is less of an issue with large and therefore longer-lasting saccades, but challenging in tasks like reading (Rayner, 1975; Slattery et al., 2011), where saccades can be as short as 20 ms. Furthermore, some paradigms even require the presentation of multiple stimuli (e.g., Buonocore et al., 2020) or entire motion sequences (Schweitzer & Rolfs, 2021) during a saccade. Trials with delayed display updates not only are lost for the analysis, but also cause oculomotor disruptions and conscious awareness of the change (Slattery et al., 2011).

Saccade-contingent stimulation has traditionally been performed with CRTs, although their phosphor persistence has led to some spurious findings (Bridgeman & Mayer, 1983; Jonides et al., 1983; W. Wolf & Deubel, 1993). More recently, high-speed projectors have been used for this purpose (Schweitzer & Rolfs, 2020; see also Richlan et al., 2013), but these systems are still prohibitively expensive for most researchers. The good temporal properties of modern gaming OLEDs should make them attractive for saccade-contingent experiments. Here we tested which change latencies could be achieved by linking the OLED with a 1000 Hz eye-tracker. Specifically, our goal was to present a stimulus exclusively during a saccade, but not before or after, in order to measure the brain-electric responses (not reported here) to these intra-saccadic stimuli.

## Methods

### Display properties and settings

**Fig. 1:**
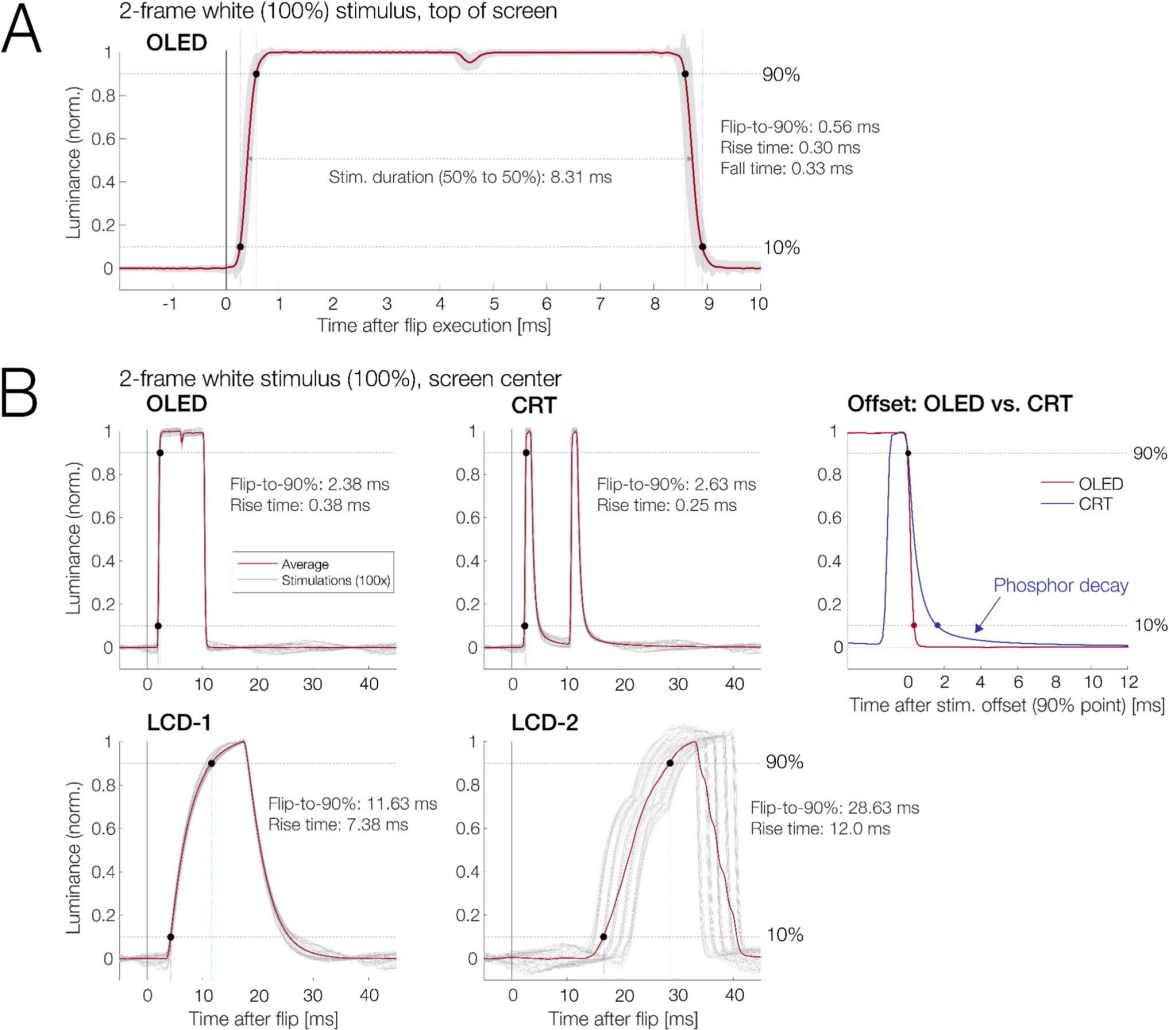
Luminance response to a white-on-black stimulus presented for two frames. In most plots, time zero marks the execution of the flip command, and the red line is the average response to 100 presentations. Black dots mark the latencies at which the signal reaches 10% and 90% of the desired luminance. **A** Enlarged view of the OLED’s luminance response. For this plot, responses were measured at the top of the screen. Gray shading indicates the region between the 2nd and 98th percentiles of the 100 presentations. The slight dip in luminance between the two frames reflects the OLED panel’s TLM flicker (see Fig. 3). **B** Comparison of the responses of different monitors to the same stimulus, but now presented in the screen center. Gray lines indicate individual responses. The OLED’s response latencies were comparable to those of a CRT monitor, but the OLED did not show phosphor persistence at stimulus offset. Response latencies were far superior to those of the two tested LCDs

**Fig. 2:**
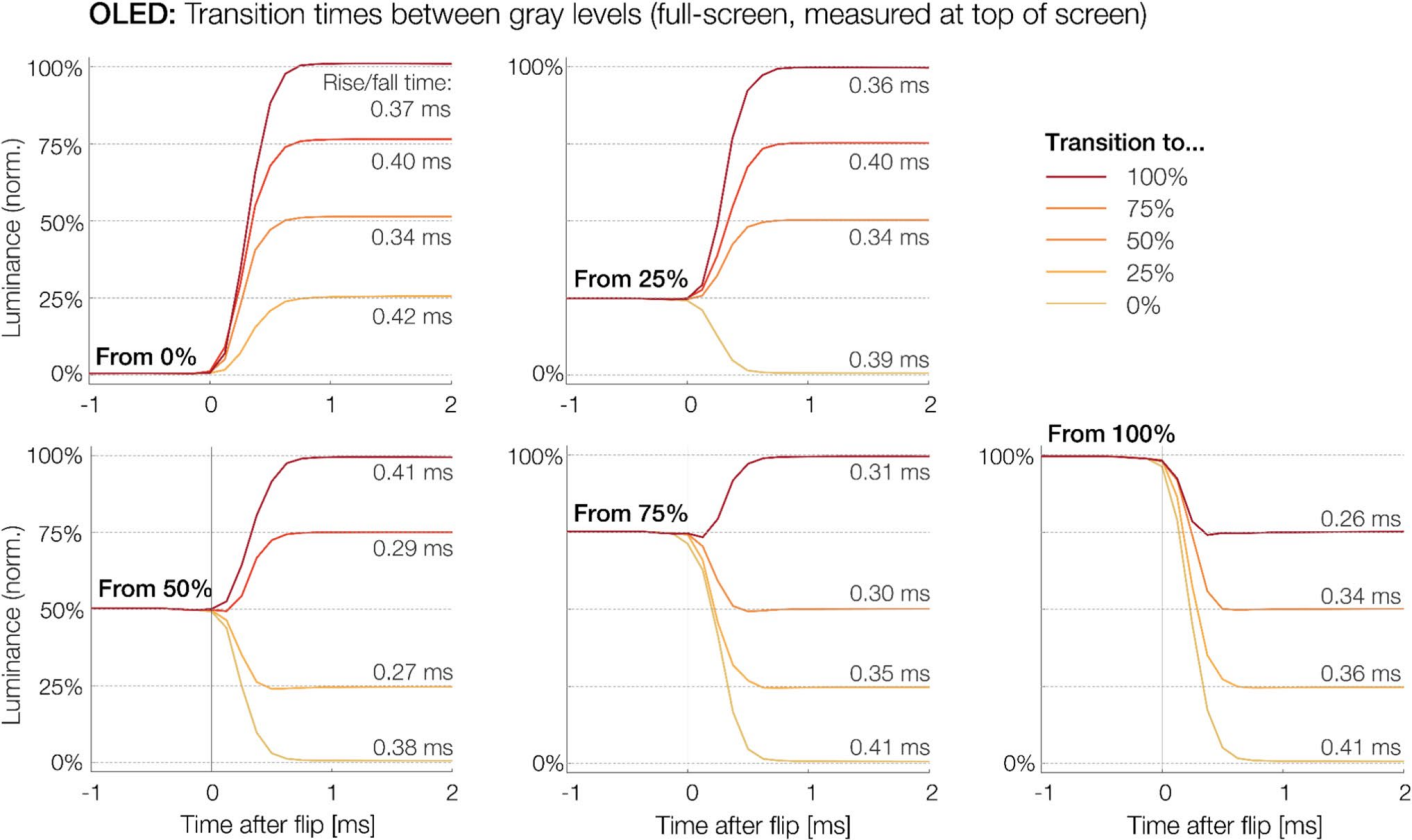
The OLED’s temporal responses for transitions between different full-screen gray levels. Each trace reflects the average of 30 measurements. The rise or fall time is reported next to each trace. They never exceeded 0.5 ms (maximum: 0.42 ms) for any transition

**Fig. 3:**
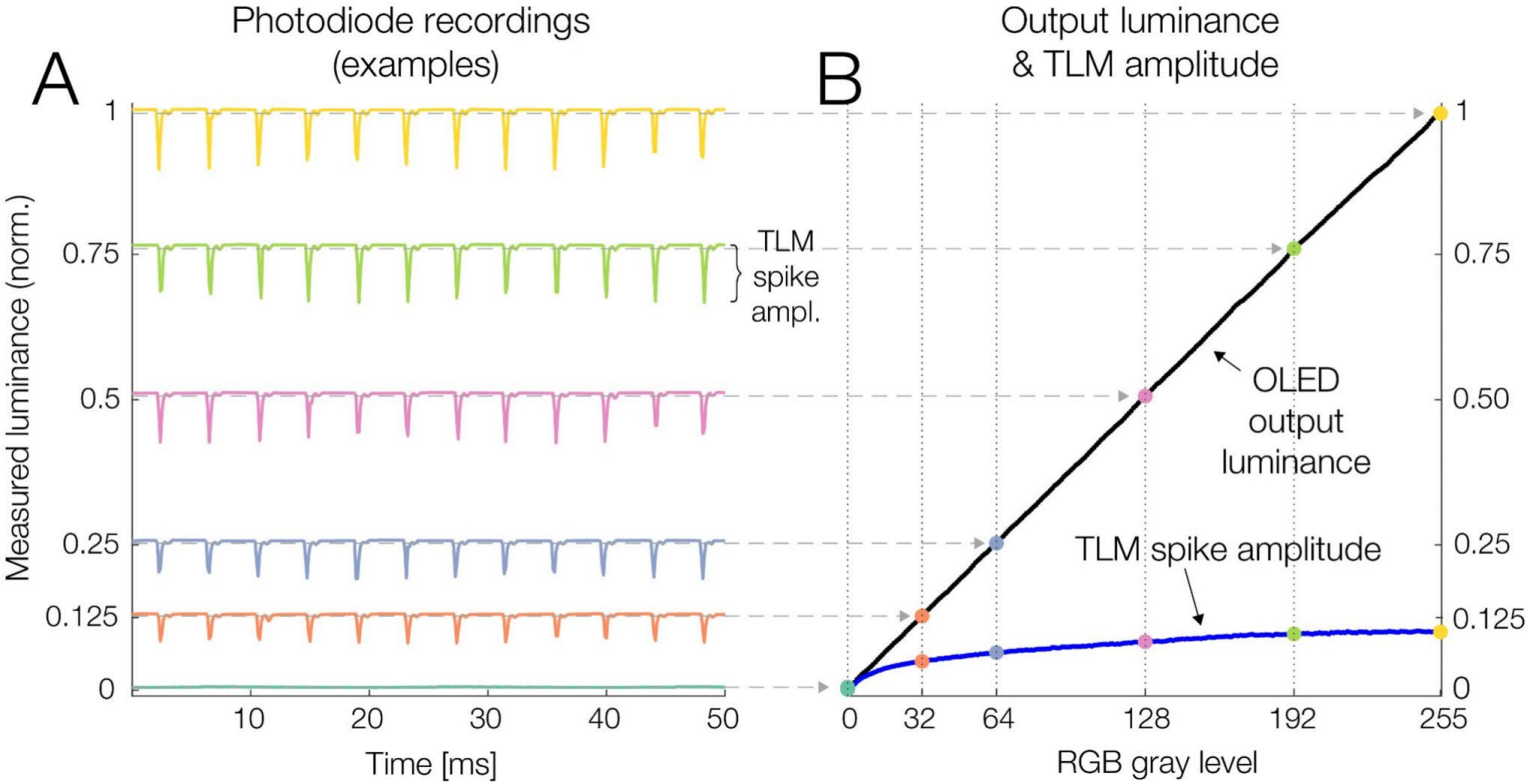
The OLED shows a temporal light modulation (TLM), a brief dip in luminance, during each refresh cycle. **A** Exemplary photodiode waveforms for full-screen 8-bit gray levels of 0, 32, 64, 128, 192, and 255. **B** Overall luminance output and TLM spike amplitude at every gray level between 0 and 255. The exemplary luminance levels shown in panel **A** are highlighted by colored dots. With increasing driving level, the monitor’s output luminance increased in a strictly ordinal manner. The amplitude of TLM flicker spikes also increased with luminance, but in a nonlinear fashion. However, because spikes are so brief, they barely affect overall luminance during a frame

**Fig. 4:**
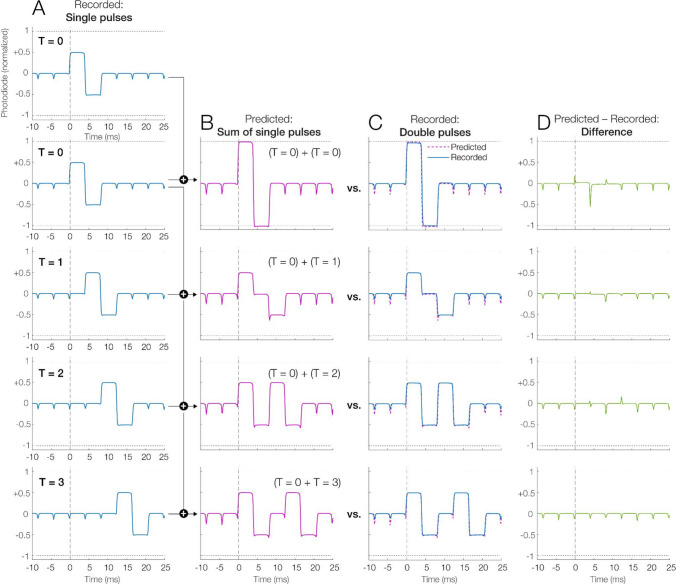
Paired-pulse test, measuring temporal dependencies of the OLED. **A** Single biphasic pulses were recorded at different temporal delays *T* (0, 1, 2, or 3 frames) relative to a trigger event sent at time 0. **B** Linear prediction: To simulate an “ideal” linear prediction, two of the single pulses from column **A** were summed, with the second pulse happening at a varying delay (*T* = 0, 1, 2, 3) after the first. **C** Recorded double-pulse patterns. For reference, the linear prediction from panel **B** is also included as a dashed pink line. **D** Difference between linear prediction and recorded double pulses. This residual error was small and partially reflected differences in the amplitude of TLM spikes

We tested three different units of the same ASUS ROG Swift OLED PG27AQDM monitor; all were purchased as part of the same shipment on the open market. The monitor features a matte 27-inch (59.0 × 33.4 cm) WOLED (white OLED) sample-and-hold display with a maximum refresh rate of 240 Hz. We will refer to this monitor as simply “OLED” in the following, or OLED-A (default unit for tests), OLED-B, and OLED-C when referring to a particular unit. In all tests, the monitor was controlled via the DisplayPort connector and operated at its native resolution of 2,560 × 1,440 pixels (see Table 1). Tests were conducted in the monitor’s standard dynamic range (SDR) mode and with its default “racing mode” activated. The monitor’s firmware version was MCM104. Features to mitigate burn-in risks (“screen move,” “pixel cleaning,” “screen saver,” “adjust logo brightness”) were switched off in the on-screen display. If not stated otherwise, the OLED was tested with its uniform brightness (UB) feature activated. This mode is intended to avoid luminance changes due to ABL. For most tests, the monitor was used at its factory gamma setting of 2.2. For certain tests (those reported in Figs. 1A, 2, 3, 4, 8B/C, 9, and 10), it was linearized to a gamma of 1 using either spot photometer measurements or a Datacolor Spyder 4 colorimeter. The variable refresh rate feature was deactivated for all tests.

**Table 1:** Comparison of monitors

Abbrev.	Manufacturer	Model	Panel type	Year	Refresh rate (Hz) (tested)	Refresh rate (Hz) (max.)	Diameter and native resolution	Hardware settings Brightness/Contrast
OLED	ASUS	PG27AQDM (Units A, B, C)	OLED	2023	240	240	27" 2,560 × 1,440	100% (260 cd/m^2^)/80%
LCD-1	ASUS	VG279Q	IPS	2020	144	144	27" 1,920 × 1,080	45% (260 cd/m^2^)/80%
LCD-2	Iiyama	Prolite G2773HS	TN	2013	100	120	27" 1,920 × 1,080	100%/50%
CRT	Iiyama	MA203DT-D VisionMaster Pro 513	CRT	2003	120	180	22" (tested at 1,024 × 786)	100%/100%

### Stimulus presentation

Stimuli were presented using Psychtoolbox-3 (PTB; Kleiner et al., 2007) under MATLAB R2023b on a 64-bit Windows 10 Enterprise computer equipped with a mid-range GPU (AMD Radeon RX 6400; controlled by AMD video driver version 31.0.23013.1023). In all tests, a TTL (transistor–transistor logic) trigger was sent from the stimulation computer’s native parallel port (using the “io64” MATLAB utility) to the bioamplifier recording the photodiode signal immediately after the execution of the “flip” command in PTB.

The creators of PTB recommend Linux for precise stimulation. We therefore repeated some of the tests under Ubuntu Linux (version 22.04.4 LTS, running Mesa 25.0.1 graphics drivers), which ran on the same stimulation computer and with the same MATLAB version (R2023b). Since timing tests under Linux yielded results similar to those under Windows, we will only mention them in the context of the UB feature, which did not function properly under Linux.

### Recording apparatus

Luminance was measured using a Konica Minolta LS-150 spot photometer with an aperture of 1°. If not stated otherwise, the photometer was mounted on a tripod at a distance of about 80 cm from the monitor. Luminance time courses were recorded with a photodiode that was sampled at 8 kHz using an *eego mylab* amplifier (ANT Neuro), which recorded without a time constant (DC recording). The photodiode used was the Brain Products Photo Sensor (BP-240–1001), which internally uses the VBPW34SR photodiode (Vishay Semiconductors). We confirmed that the photodiode’s output scales linearly with luminance, at least within the range of luminance values encountered in the present study. High-speed videos of the monitor were taken with a Casio EXILIM EX-F1 camera at 1,200 frames/s. All monitors were allowed to warm up for at least 20–30 min before testing. The monitor’s temperature at the screen surface was measured with a Sovarcate HS090E noncontact infrared thermometer. For additional temperature measurements of the entire monitor, we used an HIKMICRO ECO-V thermal imaging camera (image resolution, 96 × 96 pixels; emissivity setting, 0.97).

### Reference monitors (CRT and LCD)

The OLED’s luminance responses were benchmarked against those of three LCD and CRT monitors available in our lab. An overview is provided in Table 1. First, we compared the OLED to a CRT monitor, the Iiyama MA203DT-D, called “CRT” in the following. This monitor is from a popular series of CRT screens (“Vision Master Pro” series; for details see Elze, 2010a, 2010b) often used in time-critical studies on visual perception (e.g., Dimigen et al., 2012; Slattery et al., 2011; Wichmann et al., 2010).

Second, we compared the OLED to two LCDs of the same 27-inch diameter. The first, the ASUS VG279Q, called “LCD-1” in the following, is a fairly recent 144 Hz gaming monitor based on an in-plane switching (IPS) panel. The second, the Iiyama Prolite G2773HS, called “LCD-2” in the following, is an older 120 Hz model featuring a twisted nematic (TN) screen. All reference monitors were operated at their factory defaults for contrast and gamma (2.2) values. Where possible, their brightness setting was adjusted so that peak luminance matched that of the OLED (i.e., about 260 cd/m^2^); see Table 1. The CRT, LCD-1, and LCD-2 monitors were connected via VGA, HDMI, and DVI connectors, respectively.

### Transition times

To obtain temporal response profiles for all monitors with the photodiode, a full-white square (RGB = 255, 255, 255; size: 152 × 152 pixels) was presented either near the top (70 pixels from the upper edge; Fig. 1A) or in the center (Fig. 1B) of an otherwise black screen (RGB = 0, 0, 0) for two frames. This stimulus duration of two frames allowed us to observe both the onset (here, black-to-white) and offset response (here, white-to-black) as well as any between-cycle fluctuations. Depending on the monitor, the two frames corresponded to durations between 8.33 ms (at 240 Hz) and 20 ms (at 100 Hz). For most plots, luminance was normalized to a range of 0 to 1 (maximum), individually for each monitor. We focus on two latency measures, determined using MATLAB scripts: The monitor's rise time (or onset transition time) was defined as the interval from the stimulus reaching 10% until reaching 90% of its peak luminance (following Elze et al., 2013) in the averaged response (see Fig. 1). The flip-to-90% time was defined as the interval between the trigger marking the execution of the flip command until the moment at which luminance reached 90%.

For LCDs, transition times vary as a function of the size and direction of the luminance step between subsequent stimuli (Elze & Tanner, 2012). For the OLED, we therefore also measured rise and fall transition times between various intermediate luminance levels. For this, we presented two stimuli (L1 and L2) in immediate succession, each lasting two frames (8.33 ms). Both L1 and L2 showed one of five possible gray levels (0%, 25%, 50%, 75%, 100%; see also Elze & Tanner, 2012; Hallum & Cloherty, 2021), and we measured transition times between all 20 possible S1-to-S2 transitions (0% to 25%, 25% to 0%, 0 to 50%, etc.). Before S1 and following S2, a full-black screen (0%) was shown for 50 frames. Each S1-to-S2 transition was recorded 30 times. Transition times were determined in the averaged traces.

### Temporal light modulation (TLM) during each cycle

During each refresh cycle, the OLED exhibits a brief dip in luminance, which repeats at the refresh rate of 240 Hz. In the following, we will refer to this intrinsic display flicker as *temporal light modulation* (TLM), to distinguish it from the intentional 60 Hz flicker stimulation implemented in the high-frequency flicker experiment (reported further below). To quantify the dependence of the TLM on stimulus properties, the photodiode was placed on the center of the gamma-linearized OLED. We then presented full-screen stimuli at all possible RGB gray levels (from 0 to 255) for 50 cycles each. For each gray level, we measured (1) the monitor’s mean luminance output (mean of all photodiode samples for that level) and (2) the amplitude of the TLM spike (cf. Figure 3A), which was defined as the difference between the first and 99th percentile of all photodiode samples for that level.

### Temporal independence (paired-pulse paradigm)

As mentioned, LCDs often exhibit poor temporal independence (Elze & Tanner, 2012; Hallum & Cloherty, 2021), as their luminance depends not only on the current input but also on the immediately preceding frame. To quantify the temporal dependencies of the OLED, we used a so-called paired-pulse paradigm, following the procedures in Hallum and Cloherty (2021). This paradigm tests whether the response to a brief biphasic luminance change (i.e., a “pulse”) is influenced by the presentation of a preceding pulse; specifically, it asks whether the response to these two subsequent pulses, separated by a delay *T*, matches the linear sum of the corresponding single-pulse responses when they are shown in isolation. For this test, we recorded photodiode responses (at 8 kHz) from the center of the gamma-linearized OLED while presenting the pulses on a mid-gray background (50% luminance, RGB 128).

In a *single-pulse condition*, we first recorded the response to single pulses, as shown in Fig. 4A. These single pulses consisted of a two-frame biphasic modulation: one frame at 75% luminance followed by one frame at 25% luminance (total duration of 8.33 ms at 240 Hz). We then recorded these single pulses at four different delays (*T* = 0, 1, 2, or 3 frames) relative to a fixed trigger event.

In a *double-pulse condition*, we then recorded the response to double pulses, which physically combined a first pulse, which started at *T* = 0, with a second, delayed pulse, which started at *T* = 0, 1, 2, or 3. Ideally, with high temporal independence, these two luminance pulses should add up in a purely linear manner. For example, in the case of *T* = 0 (no time delay between pulses), the luminance modulations of both pulses should add constructively, resulting in a target sequence of a 100% luminance frame followed by a 0% luminance frame, as illustrated in Fig. 4B. At *T* = 1, the positive phase of the second pulse overlaps with the negative phase of the first, which should ideally lead to partial cancellation (to mid-gray, see Fig. 4B).

To quantify temporal dependencies, we then compared the actually recorded double-pulse waveforms (Fig. 4C) with a linear prediction (Fig. 4B) generated by summing two of the recorded single-pulse waveforms (after subtracting the 50% gray baseline) at the given delay *T*. Temporal dependencies (nonlinearities) are then evident in the residual error (linear prediction minus measurement), as plotted in Fig. 4D.

To quantify the residual error relative to the intended waveform, we used an area-based measure, which was computed separately for each delay *T*. For each *T*, within a *T*-specific window (lasting from the first luminance transition to the end of the predicted response for that delay), we subtracted the 50% gray baseline from the prediction and the measurement, formed the residual, and expressed the error as the absolute area of this residual divided by the absolute area of the prediction. This result is reported here as a percentage, with lower percentages indicating a closer agreement with the prediction and thus better temporal independence.

### Spatial uniformity

**Fig. 5:**
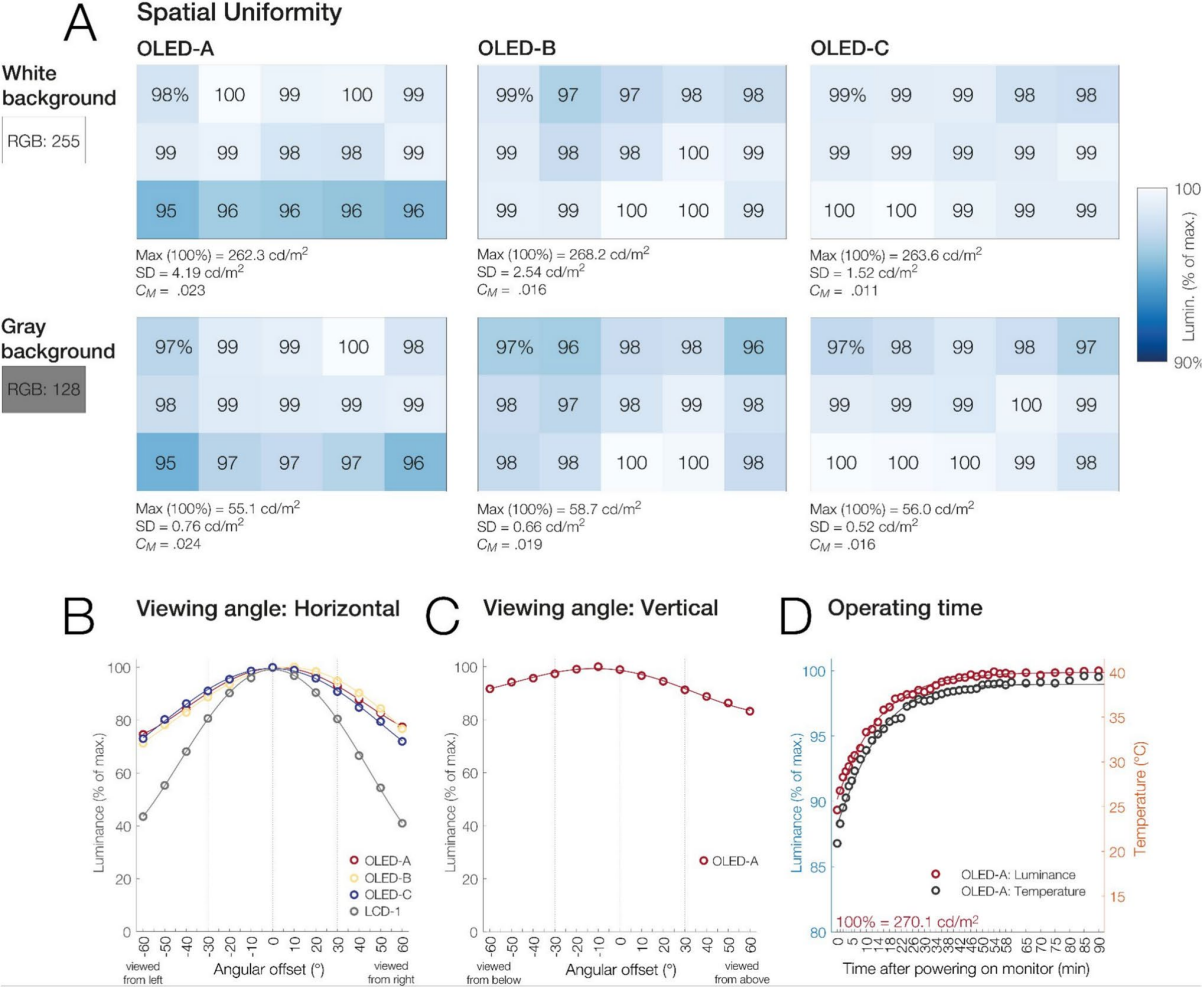
OLED luminance variation as a function of screen region, viewing angle, and operating temperature. **A** All three units of the OLED showed excellent luminance uniformity (all Michelson contrasts ≤ 0.024) with both white and gray backgrounds. **B** For all OLED units, luminance decreased by about 9% for viewing angle offsets of ± 30° (dotted vertical lines). For comparison, the decrease was about 20% for LCD-1, a monitor with an IPS panel by ASUS. **C** Vertical viewing angles, measured here only for OLED-A. There was marked vertical asymmetry, with higher luminance when the panel was viewed from below rather than above. **D** Luminance increased over time while the monitor warmed up until it reached an asymptotic luminance of 99% after 36 min

A vision science monitor must exhibit spatial uniformity, meaning that image properties should be similar at different locations. To assess luminance uniformity, we measured luminance with the spot photometer at the center of 15 screen locations arranged in a 5 × 3 grid (Fig. 5). Measurements were taken while the OLED displayed a full-white (RGB level, 255) and a mid-gray (RGB level, 128) background. For this test, the monitor was operated at its factory gamma setting of 2.2, meaning that the gray background corresponded to an expected luminance of 21.8% (0.5^2.2^) relative to the white level.

We took five repeated measurements from each location of each of the three OLED units. Display inhomogeneity was quantified by first averaging across the five repeated measurements per unit. For each unit, we then report the SD and the Michelson contrast (*C*_*M*_) across the 15 screen locations.

Finally, to determine whether spatial inhomogeneities were systematic, we compared the 5 × 3 luminance map measurements both within and between units using an inner-product permutation test. For this test, the data for the five repeated measurements were normalized independently, such that the brightest of the 15 locations of each measurement was defined as 100%. The observed similarity between two uniformity maps was defined as the mean inner product across all 25 pairwise combinations of the five repeated measurements per unit. Between-unit comparisons tested whether different monitors showed similar spatial patterns, while within-unit tests assessed measurement reliability. Statistical significance was determined by randomizing the spatial order of the 15 locations across 10,000 random permutations. For within-unit tests, locations were permuted independently across repeats; for between-unit tests, the same permutation was applied to all repeats of one unit.

### Viewing angle

To measure luminance as a function of horizontal viewing angle, the monitor was placed on a rotating turntable while displaying a full-screen white (RGB level, 255) background and then repeatedly rotated to different horizontal angles (azimuth) spanning from − 60° to 60°. The photometer, fixed on a tripod 100 cm away, was used to take measurements at the screen center for each angle. Similarly, for measurements of vertical viewing angle (elevation), the monitor was swiveled to portrait mode (i.e., pivoted) and then again rotated on the turntable from − 60° to + 60°. Measurements were again conducted for the three different OLED units, and each angle was measured three times for each unit. For comparison, a single set of horizontal viewing angle measurements was also taken for LCD-1. We did not test for shifts in color as a function of viewing angle.

### Auto-brightness limiting

We used a common procedure to assess the monitor’s ABL behavior and to test the monitor’s UB feature that is intended to prevent luminance changes as a function of the APL. To manipulate the APL, a full-white rectangle was presented in the center of an otherwise black screen (RGB level, 0). In subsequent measurements, the size of the rectangle was iteratively increased so that its area subtended 1%, 2%, 5%, 10%, 25%, 50%, 75%, or a full 100% of the screen area. In all conditions, the stimulus fully covered the field of view of the spot photometer. Each APL level was displayed for 10 s, and a photometer reading was taken from the screen center, both immediately after stimulus onset (initial luminance) and about 3–5 s later (sustained luminance). The test was conducted both without and with the UB feature activated. Additionally, to capture a phenomenon that we will refer to as “dynamic dimming” below, we repeated the test with time-resolved photodiode recordings.

### Operating time and initial warm-up

In a first step, we generally assessed how the OLED’s luminance depends on its operating temperature by testing how long it takes the monitor to reach its asymptotic luminance after switching it on. To ensure accuracy, this test was conducted after the monitor had cooled to room temperature (19.8 °C) overnight. After turning on the monitor, spot photometer and infrared thermometer readings were taken at regular intervals (initially every minute, later every 2 min, then every 5 min) from the screen center, while it continuously displayed a full-screen white background (RGB 255; 100% brightness; UB setting activated). To visualize the relationship, logistic functions were fitted to the temperature and luminance curves (Fig. 5D).

### Slow temperature–luminance interactions

In a next step, we investigated more generally how the OLED’s temperature interacts with luminance during normal operation. During our tests, we had unexpectedly observed that when a bright stimulus was displayed for long periods (several minutes), the screen not only became warmer, but this warming also caused the luminance of a subsequently presented stimulus to be slightly higher. Conversely, during prolonged presentations of a dark stimulus, the panel cooled down, leading to a slight darkening of any subsequent stimulus. As we show below, these warming and cooling effects are spatially specific. In other words, on long time scales, temperature–luminance interactions can produce a previously undocumented form of image persistence on OLEDs. To understand these luminance artifacts, we recorded photodiode, photometer, thermometer, and thermal imaging data from the monitor.

In a first test, we alternated the full-screen background of the OLED between white (RGB 255) and gray (RGB 128) every 5 min for three cycles (i.e., 30 min recording duration). During this test, we measured the panel’s surface temperature (infrared thermometer) and its luminance at the screen center (spot photometer) every 60 s.

In a second test, we displayed for 60 s a full-screen half-black/half-white pattern (RGB 0 and RGB 255), shown in Fig. 8B. Afterwards, this high-contrast stimulus was replaced by a full-screen uniform gray background (RGB 128), which remained on screen for several minutes. At various delays after the offset (*t* = 0 s) of the black/white pattern, we then measured the luminance of the gray background both in the center of the left half (formerly black) and in the center of the right half (formerly white) of the now uniformly gray screen. We also took regular thermal (infrared) images of the entire monitor (see Fig. 8B).

Finally, in a third test, we investigated the spatial specificity of these luminance-induced temperature differences. For this, a 16 × 9 black-and-white checkerboard (check size, 160 × 160 pixels; RGB 0 vs. RGB 255) was shown for 60 s. This stimulus was then again replaced by a uniform gray background (RGB 128). Thermal images were taken approximately every 15 s after checkerboard offset.

### Practical test 1: Fast flicker stimulation

We conducted two practical tests of the OLED in real-world time-critical paradigms. In a first test, we assessed whether the response times of the OLED support the faithful presentation of fast flicker time series, similar to those used in the RIFT paradigm (see also Dimigen et al., 2026). Utilizing the 240 Hz refresh rate, we presented a circular flickering patch (a disc with tapered edges) on a gray (50%) background at a frequency of 60 Hz. To reduce flicker visibility, the time series included two intermediate gray states within each four-frame cycle (i.e., patch luminance was 0%, 50%, 100%, 50%, etc.). The key question was whether the OLED could reach the intended luminance values with high fidelity within each 4.17-ms frame. To quantify the fidelity of the recorded RIFT stimulus, we calculated the coefficient of determination (*R*^2^) between the recorded flicker time series and an ideal square wave response (blue line in Fig. 9).

### Practical test 2: Intra-saccadic stimulation

To illustrate the advantages of the monitor in another time-critical scenario, we implemented a gaze-contingent display change paradigm with intra-saccadic stimulation. The goal of this simple experiment was to present a stimulus exclusively for the duration of a brief saccadic eye movement.

The experiment is illustrated in Fig. 10A. Each trial started with a white fixation cross displayed on the left side (−10° eccentricity) of an otherwise uniform gray screen. Once a fixation was registered, a second white fixation cross—the saccade target—appeared at +10° eccentricity to the right, thereby triggering the participant to execute a 20° rightward saccade. Only during the saccade, once the movement was detected online by the eye-tracker (see details below), a circular grating of varying orientations appeared in the screen center. The offset of the grating was initiated once the gaze crossed an invisible vertical boundary placed 4° (210 pixels) to the left of the saccade target. Participants then judged the grating’s orientation with a button press.

A desktop-mounted EyeLink 1000 eye-tracker (SR Research), controlled by the EyeLink extension for PTB (Cornelissen et al., 2002), recorded gaze position from the left eye at 1,000 Hz. To reduce processing delays, the EyeLink’s online filters were deactivated for gaze samples streamed to the stimulus computer via the Ethernet cable.

The photodiode was placed in the screen center, at a position that always became illuminated when the grating appeared. Data were recorded from one trained observer (one of the authors), who performed 200 trials. For eye comfort, the monitor was operated at a brightness setting of 40% for this test. For all critical events within each trial (defined in the next paragraph), a parallel port trigger was sent from the stimulus PC to both the eye-tracker and the photodiode’s bioamplifier. Offline, the photodiode data (downsampled to 2 kHz) was synchronized with the eye-tracking data using the EYE-EEG toolbox for EEGLAB (Dimigen et al., 2011) based on shared trigger pulses sent to both EyeLink and the amplifier. The average synchronization error, computed based on the alignment of triggers after synchronization, was 0.38 ms.

For each trial, we assessed the latency of six critical events. The first was the onset of the saccade (*t*_saccOn_), as detected offline after the experiment. This event reflects the moment when the eye exceeds a threshold velocity in the eye-tracking data. Offline saccade detection was performed with the widely used Engbert and Kliegl (2003) algorithm with typical parameters (velocity threshold, λ = 6 median-based SDs, min. saccade duration 8 ms).

The second latency, *t*_saccDetect_, defined the moment that the saccade was detected online using the method of Schweitzer and Rolfs (2020). Their algorithm, available as compiled C-code (https://github.com/richardschweitzer/OnlineSaccadeDetection), is a variant of the Engbert and Kliegl (2003) algorithm optimized for online use. Beginning with the presentation of the saccade target, new gaze position samples were retrieved every millisecond from the EyeLink and passed to the function together with all previous trial samples. A saccade was detected online once eye velocity exceeded λ = 9 median-based SDs for two consecutive samples (2 ms) and if the movement had an angular orientation to the right (± 45°).

The third latency, *t*_flip_, marks the moment the flip command was executed in PTB, which exchanges the GPU’s front and back buffers. The fourth latency, *t*_stimOn_, indicates the actual stimulus onset in the photodiode, defined as the moment when stimulus luminance exceeded 90% of its peak luminance. The fifth latency, *t*_StimOff_, marks the stimulus offset, defined as the latency at which luminance dropped again below 10% of its peak level. The final latency was the end of the saccade, *t*_SaccOff_, as detected by the offline saccade detection algorithm.

We focus here on several critical intervals defined by these events:

1. *saccOn* to *saccDetect*
2. *saccDetect* to* flip*
3. *flip* to *stimOn*

The latter two intervals depend strongly on the monitor’s properties: Interval (2) is largely determined by the monitor refresh rate because it influences when the next flip can happen. Interval (3) is influenced both by the refresh rate (because it determines how long it takes for the screen's vertical refresh to arrive at the screen center) and by the monitor’s transition time.

These intervals can also be combined into (4) the *display change latency* (as commonly reported, for example, in reading research), which is the sum of (2 + 3). Finally, (5) *total latency* was defined as the entire interval from saccade onset to stimulus onset (1 + 2 + 3).

## Results

### Transition times

Figure 1A shows the average response of the OLED to 100 presentations of a white stimulus. The stimulus was shown for two frames, corresponding to a nominal stimulus duration of 8.33 ms (two frames of 4.17 ms each). For Fig. 1A, the stimulus was presented near the top of the screen. Time zero marks the timing of a trigger sent at the execution of the flip command.

Both onsets (black-to-white transitions: rise time) and offsets (white-to-black transitions: fall time) were extremely fast: They lasted 0.3 ms (rise) and 0.33 ms (fall) and were almost perfectly symmetrical. Relative to the flip, the stimulus reached 90% of its peak luminance after about half a millisecond (0.56 ms). Between the two display frames, there was a dip in luminance by about 5% in the averaged trace. This dip reflects the TLM flicker at the 240 Hz refresh rate (see next section). Otherwise, the OLED’s luminance response was close to a square wave (Elze et al., 2013; Matsumoto et al., 2014). The horizontal arrow in Fig. 1A highlights the interval between the 50% luminance points, which were 8.31 ms apart. The monitor’s duty cycle therefore closely matched the nominal stimulus duration of 8.33 ms.

Figure 1B compares the OLED’s luminance response to that of selected CRT and LCD monitors. For Fig. 1B, stimulus and photodiode were placed in the screen center. Since the OLED updates from top to bottom, this adds some delay relative to the flip latency. As Fig. 1B shows, the rise time of the OLED (in this test about 0.38 ms) was only slightly longer than th of the CRT monitor running at 120 Hz (0.25 ms). Notably, however, the mean flip-to-90% latency was even shorter for the OLED (2.38 ms) than the CRT (2.63 ms).

In contrast to the OLED, the CRT showed asymmetric onset and offset responses with a slower fall than rise time. The rightmost panel in Fig. 1 B compares the offset responses in detail. For this panel, responses were temporally aligned to stimulus offset, the point when luminance fell below 90%. Whereas the OLED’s luminance dropped to zero within half a millisecond, the CRT showed a more gradual offset due to phosphor persistence. Specifically, after the CRT’s luminance had fallen below 10%, it took another 10 ms to decay to 1% luminance and another > 30 ms to decay entirely (to near 0%).

The bottom panels in Fig. 1B show the responses for the two tested LCDs. The results exemplify the problems seen with many LCD screens (Ghodrati et al., 2015): sluggish responses and often asymmetrical onset-versus-offset profiles. The more recent LCD monitor (LCD-1) had a rise time of 7.38 ms and reached 90% luminance 11.63 ms after the flip. The older LCD-2 showed even slower transitions and also showed synchronization issues with the flip command (visible in the single-trial response in Fig. 1B).

Figure 2 visualizes the OLED’s transition times between full-screen stimuli of various gray levels. For this test, stimuli were presented as full-screen stimuli to facilitate comparisons to recent transition time measurements reported by Haila et al. (2025). Overall, rise times (*M* = 0.36 ms) and fall times (*M* = 0.35 ms) for these full-screen stimuli were numerically slightly longer than those we measured for the smaller stimuli presented in Fig. 1A (rise, *M* = 0.30 ms; fall, *M* = 0.33 ms). Importantly, however, regardless of the size and direction of the luminance step, all transition times remained within a narrow range (0.26 to 0.42 ms) well below half a millisecond.

### Temporal light modulation (TLM)

As visible in Figs. 1A and 3, the OLED exhibits a spike-like TLM at its refresh rate of 240 Hz. In OLEDs, this TLM typically results from brief voltage changes at the pixel’s transistor gates which control the light output during each refresh cycle. This subtly alters the pixel’s brightness for a brief moment. Figure 3A illustrates the TLM at six exemplary luminance levels. Each spike lasts about 0.6 ms of the 4.17-ms frame duration.

Figure 3B compares the monitor’s overall luminance output at every gray level (from 0 to 255; averaged across time) to the size of the TLM spike. For a black screen (RGB 0), TLM spikes were absent. With increasing luminance, spike amplitude then increased in a nonlinear fashion until it reached a maximum for full-white (RGB 255, or 260 cd/m^2^).

### Temporal independence (paired-pulse paradigm)

The paired-pulse test assessed the OLED’s temporal dependencies (Hallum & Cloherty, 2021). The results are shown in Fig. 4. In this figure, panel 4 A shows the waveforms for single pulses, recorded at various temporal offsets. Importantly, we can see that the predicted luminance output from summing up two of these single pulses (Fig. 4B) closely matched the pattern seen for actual recorded double pulses (Fig. 4C), resulting in a small difference or error (Fig. 4D).

We quantified this error as the percentage of the area under the curve of the linear prediction. For the four delays, the errors were 4.59% at *T* = 0, 4.38% at *T* = 1, 3.18% at *T* = 2, and 3.08% at *T* = 3. Overall, these numbers suggest that the OLED does not suffer from significant issues with temporal dependence.

Notably, some of this error is also not due to true display nonlinearity, but explained by the simulation procedure, which summed two single-pulse recordings. This means that the display’s TLM flicker spikes present in the data are also summed, thereby artificially doubling their amplitude, even during intervals in which only a neutral gray background was presented. In contrast, genuine nonlinearities were mostly seen at *T* = 0 (see the larger spike in the residuals visible in Fig. 4D, top panel), and caused by minor differences in dark-to-bright transition times between the single-pulse transitions (where two smaller luminance steps are summed) and the actual double-pulse transition (where one larger luminance step occurs). Thus, in contrast to many LCD monitors (e.g., Hallum & Cloherty, 2021), the analysis suggests an absence of strong temporal dependencies for the OLED.

### Spatial uniformity

Figure 5A illustrates the luminance uniformity for three different units of the OLED for a white (about 260 cd/m^2^) and a gray (about 56 cd/m^2^) background. Overall, all units of the OLED showed excellent uniformity. For the white background, the Michelson contrast (*C*_*M*_) was 0.023 for OLED-A, 0.016 for OLED-B, and 0.011 for OLED-C. For the gray background, the *C*_*M*_ was 0.024, 0.019, and 0.016, respectively. Standard deviations across locations are given in Fig. 5A.

The inner-product permutation test confirmed high *within*-unit measurement reliability for all three monitors (all *p*s <.001 for both white and gray backgrounds), meaning that the five repeated measurements produced highly similar spatial maps. *Between*-unit comparisons of uniformity patterns produced mixed evidence: For the white background, spatial maps of units OLED-A versus OLED-B (*p* =.998) and OLED-A versus OLED-C (*p* =.977) did not correspond, whereas a nonsignificant trend towards a matching spatial luminance pattern was observed for OLED-B versus OLED-C (*p* =.075). For the gray background, OLED-B and OLED-C showed significant spatial similarity to each other (*p* <.01), while the other comparisons did not (both *p*s >.585). Together, our findings suggest that each unit of the monitor produces a consistent but largely idiosyncratic pattern of very weak (all *C*_*M*_ < 0.024) luminance inhomogeneity. Significant spatial correspondence was only found between two of the three units when displaying gray.

### Viewing angle

Figure 5B depicts luminance as a function of viewing angle for the three OLED units. At viewing angle offsets (azimuth) of ± 30°, luminance dropped by 8.6% to a value of 91.4% (value averaged across the three units, three measurements per unit, and across positive and negative offsets). In comparison, for LCD-1, a fairly recent ASUS gaming monitor using an IPS panel, the drop-off was almost 20% (to 80.5%, averaged across positive and negative angular offsets). At extreme offsets of ± 60°, luminance dropped on average by 25.8% (to 74.2%) for the OLEDs and by 57.8% (to 42.2%) for LCD-1.

For all three OLED units tested, there was slight asymmetry for negative (viewed from left) versus positive (viewed from right) horizontal angular offsets, which was not present for LCD-1. At ± 30°, the magnitude of this left–right asymmetry amounted on average to 3.0% luminance (OLED-A, 3.1%; OLED-B, 6.2%; OLED-C, 0.3%).

Figure 5C also shows vertical viewing angles (elevation), which were only measured for OLED-A. While viewing angle dependency was comparatively mild, there was clear vertical asymmetry, such that the screen was more luminous when viewed from below as compared to above. For example, at an offset of − 30° (from below), luminance was reduced by 2.7%, whereas at an offset of + 30° (from above), the reduction was 8.7%. Overall, the OLED shows good luminance stability across different viewing angles.

### True black

When showing a full-screen black stimulus, the OLED did not produce light emissions measurable with our photometer. Subjectively, the screen remained invisible after 15 min of dark adaptation in a totally dark room. A long exposure photograph (tripod-mounted Sony Alpha 6000 camera, Samyang 12 mm lens set to an F2.0 aperture, exposure time of 30 s at ISO 20,000) also provided no evidence of light emissions.

### Operating time

After switching on the monitor, luminance increased steadily over 90 min from an initial level of 91.6% (or 241.3 cd/mm^2^) at room temperature to the full 100% once the monitor had warmed up to 39.5 °C (Fig. 5D). An asymptotic luminance level of 99% (267.8 cd/mm^2^) was reached after 36 min of operation (at 37.6 °C).

### Auto-brightness limiting behavior

**Fig. 6:**
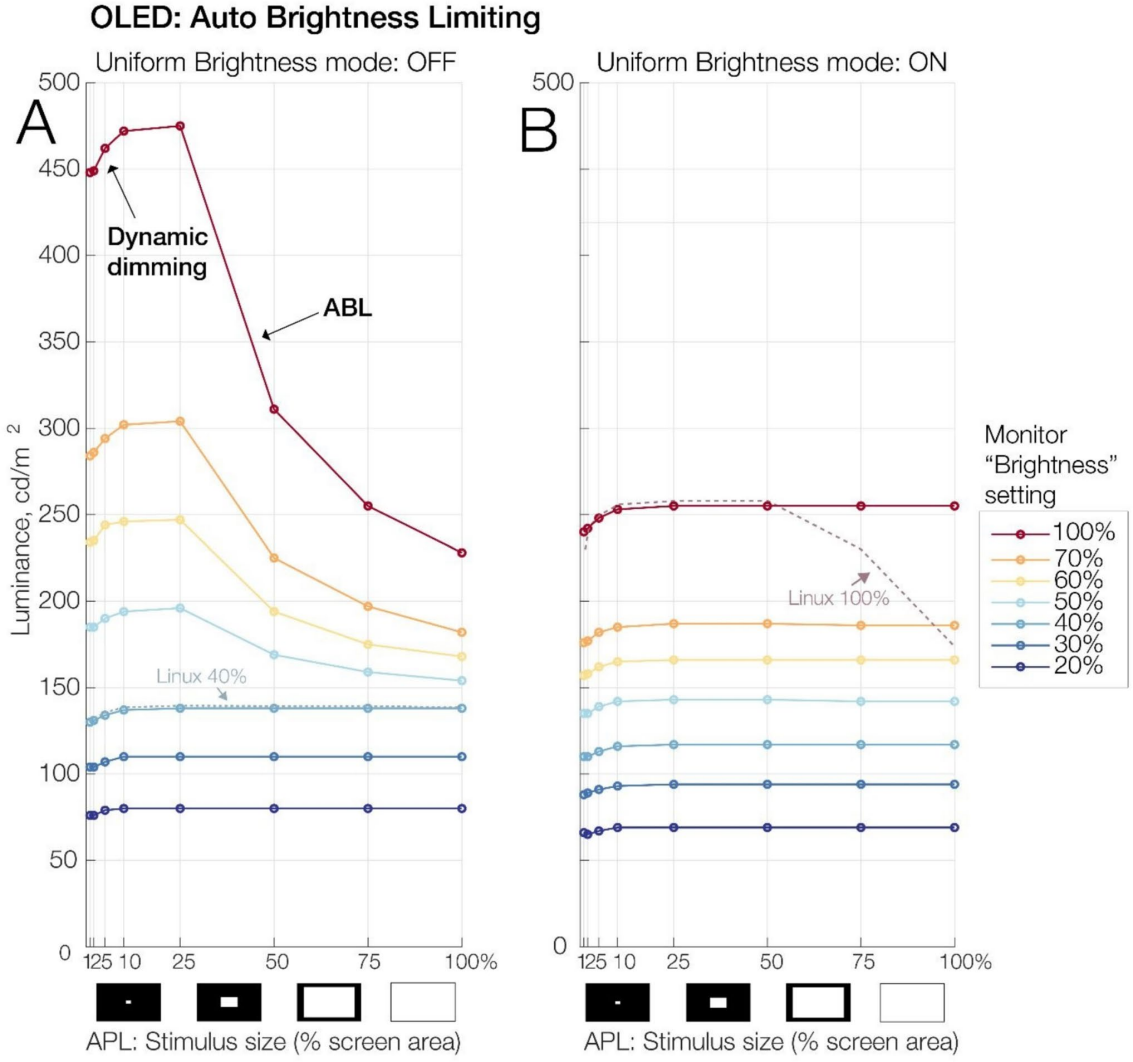
Auto-brightness limiting (ABL) behavior of the OLED as a function of the average picture level (APL), the monitor’s brightness setting, and the activation of the uniform brightness mode. The x-axis indicates the area (in % of total screen) filled by a white stimulus presented on an otherwise black background. This is also called the APL, the monitor’s overall luminance output. As can be seen, sustained stimulus luminance decreased drastically with increasing APL, a typical behavior of OLEDs. Importantly, however, ABL could be reliably prevented by operating the monitor at brightness settings of 40% or less (panel **A**) or alternatively, by activating its uniform brightness mode (panel **B**) which prevented ABL at all brightness settings. The slight decrease in sustained luminance for small bright stimuli (10% area or less) visible in all plots is caused by “dynamic dimming,” detailed in Fig. 7. In our tests, the uniform brightness feature did not work properly under Linux, as exemplified here for a brightness setting of 100% (panel **B**, dotted line). However, ABL could also be prevented under Linux by running the monitor at or below 40% brightness (panel **A**, dotted line)

The uniform brightness feature is intended to eliminate local luminance changes due to changes in the average picture level. Figure 6 shows the luminance of a white stimulus as a function of its size (i.e., the APL) both without (panel A) and with (panel B) the UB mode activated. Without the UB mode (Fig. 6A), stimulus luminance changed drastically with the APL whenever the monitor was operated at brightness settings above 40%. The 40% setting corresponds to luminance of about 140 cd/m^2^. Importantly, however, at settings of 40% or less, no ABL occurred. This suggests that ABL can be reliably prevented by operating the monitor at moderate brightness settings.

Figure 6B shows the results from the same test with the uniform brightness mode activated. At any given brightness setting, the UB mode resulted in a slight decrease in the monitor’s luminance. More importantly, however, it prevented dimming due to ABL at all brightness settings, meaning that the monitor’s brightness no longer varied according to the APL. With this setting, brightness could be safely increased to 100% (corresponding to about 260 cd/m^2^) without any measurable ABL behavior.

We also repeated this test under Ubuntu Linux. To our surprise, we found that the UB mode did not work properly under Linux. This is exemplified in Fig. 6B for the condition with 100% brightness (dotted line). This issue may be due to the specific version of Linux or graphics drivers used here, since a similar issue with Linux was not observed by Haila and colleagues (cf. Haila et al., 2025). In any case, our tests show that Linux users can always safely operate the monitor at brightness settings of ≤ 40% (see dotted line in Fig. 6A) to prevent ABL.

### Dynamic dimming

**Fig. 7:**
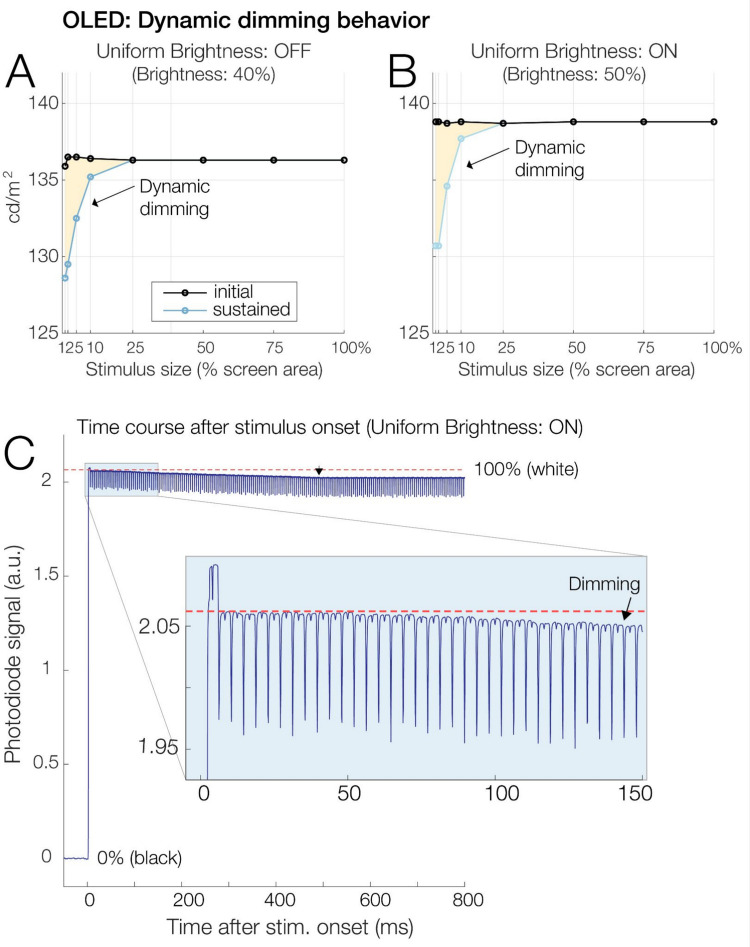
Dynamic dimming of small bright stimuli. **A** Spot photometer readings for a white (100%) stimulus of varying size presented on a black background. Readings were taken immediately after stimulus onset (“initial,” black line) and 3–5 s later (“sustained,” blue line). Interestingly, small (≤ 10% of screen area) but not large stimuli dynamically decreased in luminance by about 2–5%, with smaller stimuli experiencing stronger dimming. Notably, this happened even at low brightness settings at which ABL (see Fig. 6) no longer occurred. **B** In contrast to ABL, this dynamic dimming was seen regardless of the uniform brightness mode. **C** Photodiode time course of dynamic dimming (average of 100 stimulations) for a small (2% of screen area) white stimulus. Luminance decreased over the first ~ 500 ms and then plateaued

We observed an unexpected dynamic dimming behavior that happened exclusively for relatively small and bright stimuli (Fig. 7) and that was independent of the ABL behavior described above. Specifically, we found that the luminance of small full-white stimuli subtending 10% or less of the area of an otherwise dark screen decreased in luminance by up to 5% over the first 500 ms after stimulus onset.

The spot photometer data (Fig. 7A and 7B) show how this dimming affects only small stimuli (e.g., 2% of screen area, which lost 5% of their luminance) but not large stimuli (no change for stimuli that are ≥ 25% of screen area). Also, unlike ABL, which was effectively prevented by the UB feature, dynamic dimming occurred regardless of the UB setting (compare Fig. 7A and 7B). The photodiode recordings in Fig. 7C also revealed the temporal dynamics of this dimming behavior: Luminance decreased linearly over the first 500 ms and then plateaued.

### Slow temperature–luminance interactions

**Fig. 8:**
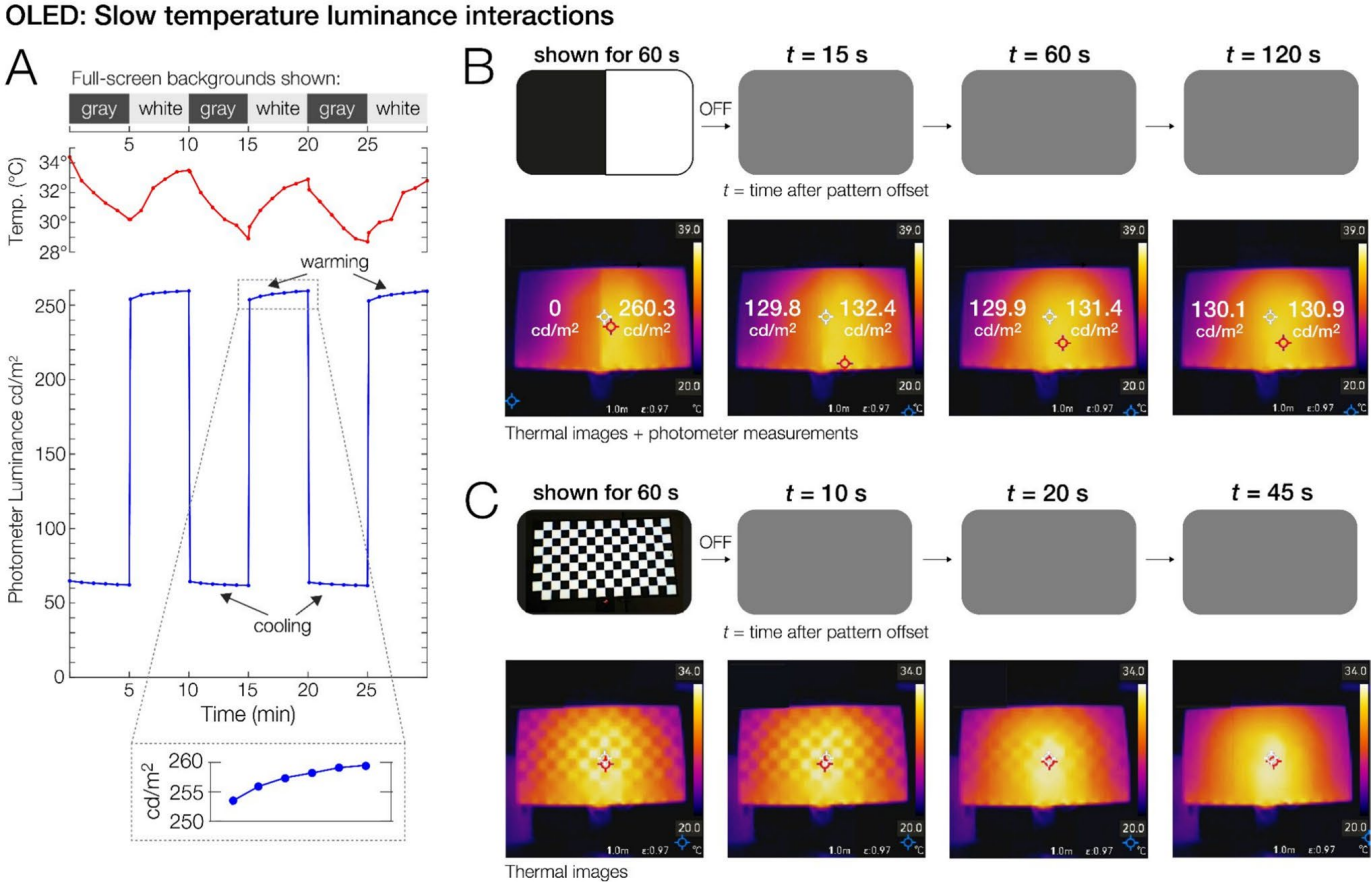
Slow temperature–luminance interactions for the OLED monitor. **A** Surface temperature (top) and luminance at the screen center while the display alternated every 5 min between full-screen white and gray backgrounds. During white presentation, the panel gradually warmed and brightened; during gray, it cooled and dimmed. **B** A half-black/half-white stimulus was shown for 60 s and then replaced by a full-screen gray background (RGB, 128). Thermal images and photometer readings (from both halves of the screen) at various delays after the offset of the black/white stimulus show that the previously white half remained warmer and slightly brighter for over a minute, despite the now neutral background. **C** After a checkerboard (160 × 160 pixels per check) was shown for 60 s, the formerly white areas remained warmer than the formerly black areas. This temperature difference took tens of seconds to fully dissipate

So far, we have mainly described the excellent performance of the OLED for short stimuli and rapid stimulus updates. However, during our tests, we also observed a novel type of temperature-mediated luminance artifact that can create luminance drifts and even a form of mild image persistence at longer time scales (tens of seconds or minutes). Figure 8 summarizes these findings.

Figure 8A shows the monitor’s temperature and luminance while the screen alternated between a white (RGB 255) and a gray (RGB 128; here, 21.8%) background. Whenever the OLED displayed the darker background, it gradually cooled and its luminance dimmed over several minutes. Conversely, whenever the background switched to white, the now cooled-down panel warmed and brightened.

Across the three gray–white cycles depicted in Fig. 8A, the average warming rate following white presentation was + 0.667 °C/min, accompanied by a luminance increase of 1.193 cd/m^2^/min. The increase was strongest within the first minute (+ 2.633 cd/m^2^) and weaker during minute 5 (+ 0.467 cd/m^2^). Once the panel switched to gray, the average cooling rate was − 1.267 °C/min, accompanied by a luminance decrease of − 0.495 cd/m^2^/min (− 0.917 cd/m^2^ during minute 1, − 0.207 cd/m^2^ during minute 5). Thus, changes in the OLED’s background luminance (or APL) cause slow but systematic drifts in luminance over extended time spans (here, a drift of up to 5 cd/m^2^ over 5 min).

Figure 8B illustrates that these temperature–luminance interactions are spatially localized and can even create a mild form of image persistence. In this test, the gamma-linearized panel showed a half-black/half-white (black, 0 cd/m^2^; white, 260.3 cd/m^2^) pattern for 60 s. Thermal images show how the white half became warmer than the black half during this time. The interesting finding is what happens after the offset of the black/white pattern, defined here as *t* = 0 s. At this time, the pattern was replaced by a uniform gray background (RGB 128, or ~ 130.5 cd/m^2^), and we took thermal images and photometer readings from both sides of the screen at regular intervals.

Measurements show how the previously white half of the panel remained hotter and brighter, whereas the previously black half remained cooler and darker, even though the same background was now shown. At *t* = 15 s after the offset, the formerly black side remained 2.6 cd/m^2^ darker than the formerly white side. The difference declined to 1.4 cd/m^2^ after 20 s and to 0.8 cd/m^2^ after 45 s. At 180 s (not shown in Fig. 8B), luminance was nearly uniform again (difference < 0.5 cd/m^2^) between the halves.

The spatially localized nature of the heating/cooling is also illustrated in Fig. 8C. Here, we presented a black-and-white checkerboard for 60 s. Thermal images taken at various delays after checkerboard offset reveal that the formerly white squares remained warmer than the formerly black squares for several tens of seconds. Only after around 45 s had these local temperature differences fully dissipated (Fig. 8C, right side).

In summary, on slow time scales, we observed that the OLED can produce a spatially blurred form of image persistence that is caused by interactions between the panel’s temperature and luminance. Specifically, when very bright or dark stimuli are presented for extended durations (i.e., tens of seconds or minutes), the local screen temperature changes, thereby producing mild but prolonged differences in luminance that are localized. For an extreme condition (a maximum contrast stimulus shown for a full minute; Fig. 8B), the resulting luminance artifacts were on the order of 2–3 cd/m^2^, corresponding to about 0.7–1.5% of the OLED’s peak luminance (around 260 cd/m^2^).

### Practical test 1: Fast flicker stimulation

**Fig. 9:**
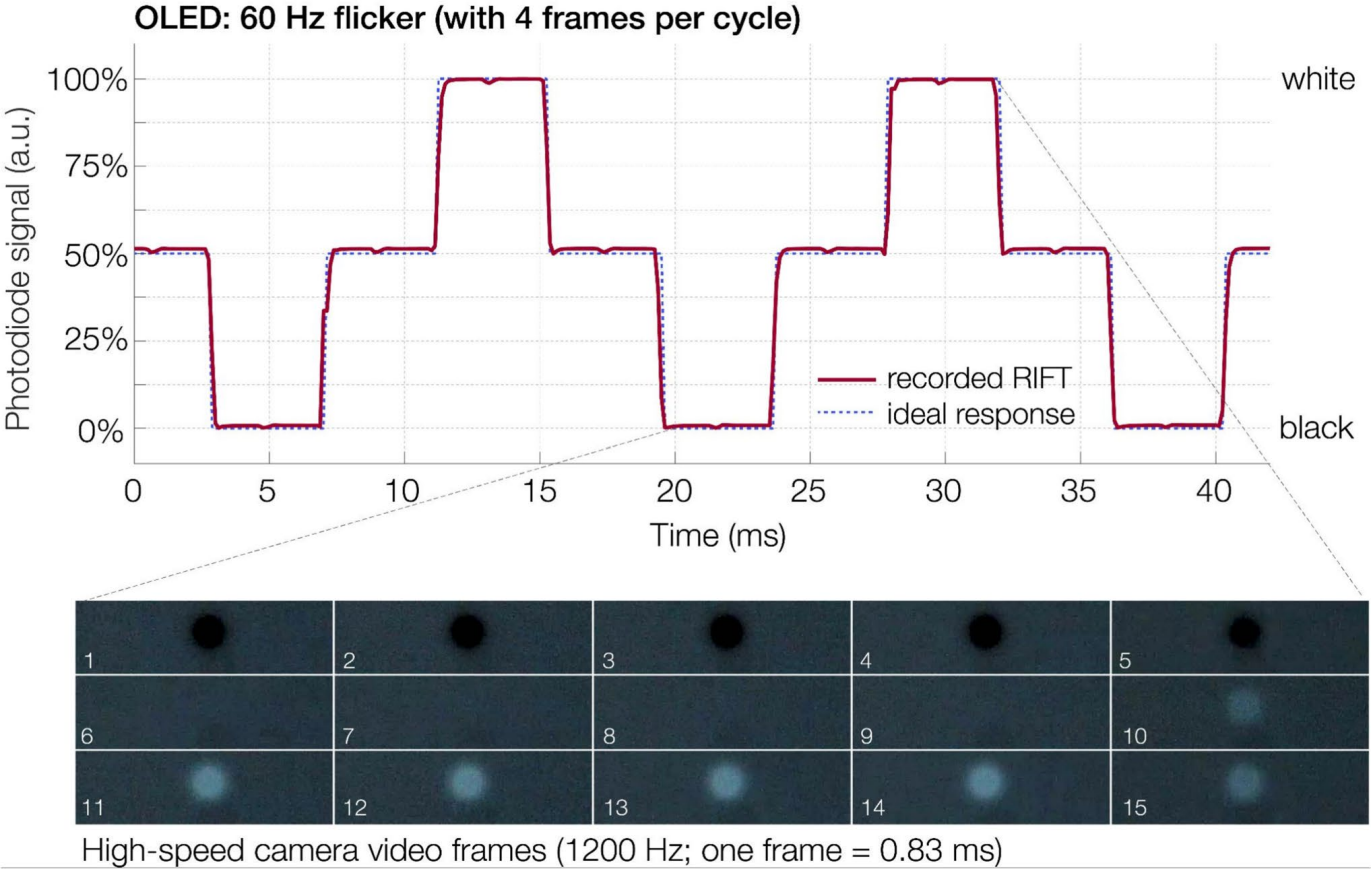
Upper panel: Luminance response for a round patch flickering at 60 Hz (background, 50%; luminance of patch = 0%, 50%, 100%, 50%, etc.) with an intermediate gray level to reduce the flicker’s visibility, as in rapid invisible frequency tagging (RIFT). The dotted blue line indicates the theoretical ideal response. Lower panel: Frames taken from a high-speed video of the flicker sequence. Each video frame lasts 0.83 ms

As a first practical test, we tested how well the OLED could display rapid stimulus sequences, where luminance changes on every frame. Figure 9 plots two cycles of a flickering patch presented at 60 Hz with four frames per cycle. Due to the rapid transition times of the OLED (of only 0.3–0.4 ms, see Fig. 1), each of the 4.17-ms-long flicker states could be faithfully presented at the intended duration, and there was a good match to a theoretical, ideal time series with instantaneous transitions that was overlaid on the recorded data (blue dotted line in Fig. 9). To quantify the fidelity of the recorded RIFT signal compared to this ideal square wave signal, we calculated the coefficient of determination (*R*^2^) between the time series for an arbitrary 1-s interval (60 RIFT cycles). The ideal square wave model accounted for 97.6% of the total variance in the recorded photodiode signal (*R*^2^), indicating a low deviation from an ideal pattern.

### Practical test 2: Intra-saccadic stimulation

**Fig. 10:**
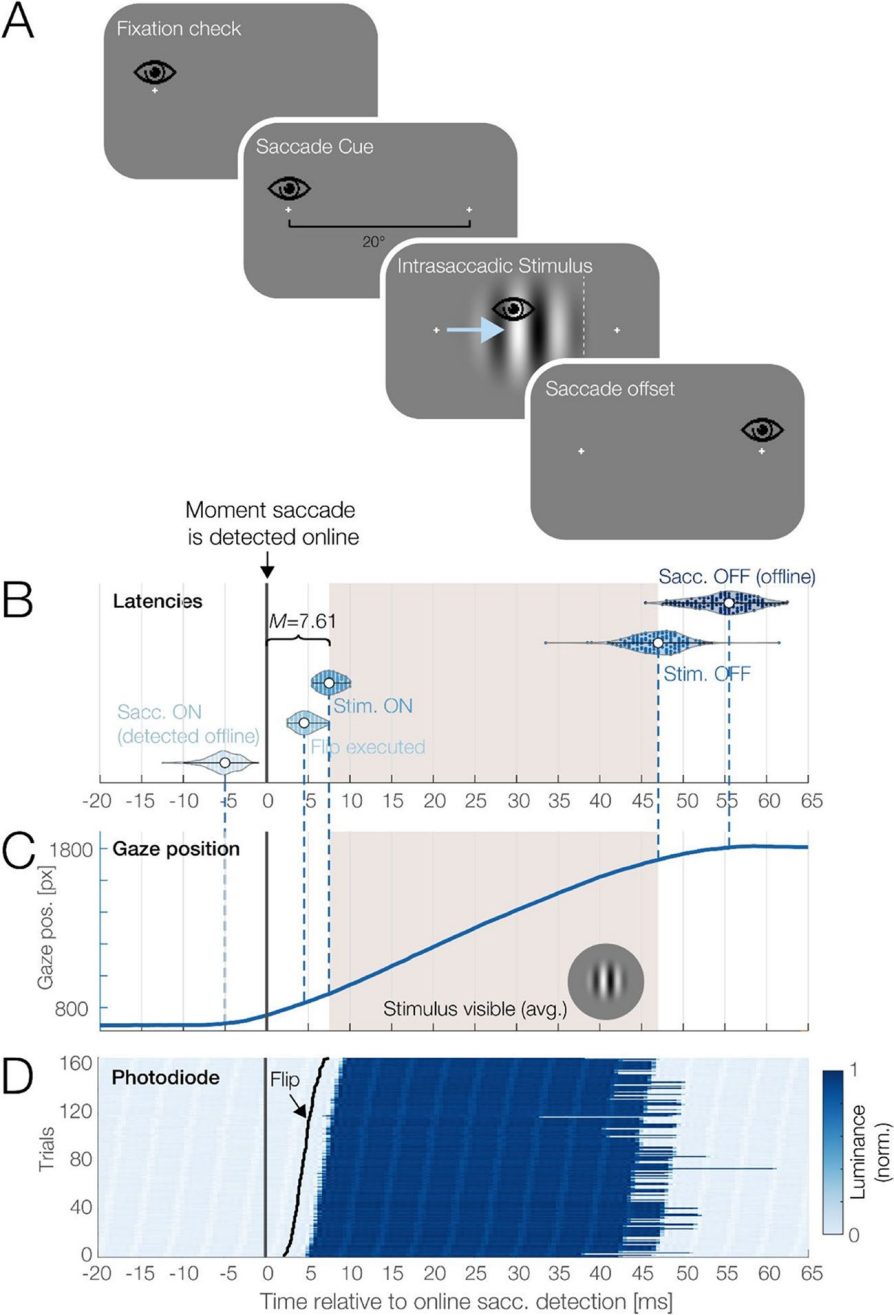
Practical speed test in an experiment with gaze-contingent stimulation. Goal was to show a grating only for the duration of a saccade, but not before or after. **A** Following a fixation check, the participant executes a 20° saccade towards a target. Once the saccade is detected with a real-time algorithm, the flip command is given and a circular grating is displayed. The offset of the grating is triggered by the eyes crossing an invisible vertical boundary to the left of the target (white dotted line). **B** Overview of critical latencies, plotted relative to the moment that the saccade was detected online (time zero). Violin plots show median values and single-trial data points for saccade onset, flip latency, stimulus onset, stimulus offset, and saccade offset. Shading indicates the average duration that the grating was visible. **C** The viewer’s average horizontal gaze position. **D** Single-trial photodiode responses, sorted by flip latency in the trial (black line). Visible stripes result from the slight OLED flicker during each refresh cycle

Our second practical test involved a time-critical display update during a saccadic eye movement. The online saccade detection led to some premature display updates before saccade onset (false alarms; 9.5% of trials), caused by microsaccades or unstable eye tracking. Trials with false alarms or other issues (e.g., eye blinks) were removed for the current analysis (total, 17% of all trials). Results for the remaining trials of one observer are visualized in Fig. 10 (using the Violinplot-Matlab package; Bechthold, 2016).

As can be seen, it was routinely possible to change the screen well before the saccade reached its peak velocity. The mean durations of the critical intervals were as follows:

1. *SaccOn* to *SaccDetect*: 5.28 ms (*SD* = 1.84 ms)
2. *SaccDetect* to *Flip*: 4.69 ms (*SD* = 1.19 ms)
3. *Flip* to *StimOn*: 2.92 ms (*SD* = 0.29 ms)

Relative to the online detection of the saccade (*SaccDetect*), the display was updated on average with a *display change latency* of 7.61 ms (*SD* = 1.18 ms). Relative to the saccade’s actual onset, as detected offline with more sensitive thresholds, the stimulus onset occurred with a *total latency* of 12.89 ms (*SD* = 2.25 ms). The average saccade duration was 60.36 ms; of this, it was possible to show the stimulus on average for 39.33 ms, or 65.16% of the saccade. Only in rare cases (5 of 200 trials), the stimulus was switched off too late, after the saccade had landed.

In summary, using the OLED, it was possible to reliably turn the stimulus on and off again during a saccade with short delays. This made it possible to display the grating for two thirds of the movement’s duration, but not before or after. By using this setup together with simultaneous EEG recordings (Dimigen et al., 2011), we can assess whether and how the intra-saccadic stimulation of the retina (Campbell & Wurtz, 1978; Nicolas et al., 2021; Wurtz, 1969) contributes to eye movement-related neural activity in the EEG (Dimigen & Ehinger, 2021). These results are reported elsewhere.

## Discussion

While the advantages of OLED panels in delivering excellent image quality are well recognized, these displays are not yet commonly used in studies on visual perception or cognitive neuroscience. In this work, we evaluated OLED technology for vision research by testing a promising model from a new generation of fast OLED gaming monitors, mainly focusing on its temporal properties for monochromatic stimuli. The monitor showed excellent performance in “fast” paradigms that involve low-latency display updates or rapid stimulus sequences. However, the panel also produced luminance artifacts when stimuli were shown for extended durations (e.g., minutes), which may critically affect some “slow” experimental paradigms.

Overall, we found that the 240 Hz monitor has excellent timing properties, consistent with the already promising results reported for much earlier OLED models (Cooper et al., 2013; Elze et al., 2013; Ito et al., 2013) as well as those recently reported by Haila and colleagues (2025), who independently evaluated the same monitor using a complementary set of performance metrics. In our tests, transition times and flip-to-90% latencies were significantly better than those of the tested LCDs and on par with those of a CRT monitor. Like a CRT monitor, the OLED was able to show true black, thereby supporting high contrasts. Unlike a CRT monitor, however, the OLED was also able to display stimuli as continuous, approximately square wave-like luminance patterns at their nominal duration (Elze, 2010b; Haila et al., 2025) and without slowly decaying offsets due to phosphor persistence. A paired-pulse test revealed that the OLED does not suffer from strong temporal dependencies as seen with some LCD panels (e.g., Hallum & Cloherty, 2021).

The monitor’s 240 Hz refresh rate supports fine-grained gradations in stimulus duration that are important for many paradigms in experimental psychology (Poth et al., 2018).^1^ The OLED’s fast transition times should also improve the measurement of visually evoked brain potentials as compared to LCDs (Husain et al., 2009; Kaltwasser et al., 2009; Matsumoto et al., 2014; Nagy et al., 2011).

### Sources of luminance variation

We identified several factors that subtly influenced the luminance displayed by the OLED. Within each 240 Hz refresh cycle, the monitor exhibited a brief temporal luminance modulation, a short dip in luminance (lasting around 0.6 ms) that is typical for OLED panels. However, due to its high frequency and short duration (see Fig. 3), this display flicker is likely imperceptible to the viewer and has barely any impact on average luminance over time.

We also assessed luminance variations due to screen location, viewing angle, and operating time. With a standard deviation between screen locations of 1.5%, the monitor’s luminance uniformity was excellent for all three tested units. The Michelson contrast across spatial locations never exceeded a value of 0.024, which is much lower than that of most consumer LCDs (Ghodrati et al., 2015).

 Whereas CRTs are barely affected by viewing angle (Brainard et al., 2002), poor viewing angles are a major drawback of LCDs. For the OLED, luminance decreased to 92% at viewing angle offsets of ± 30°, the maximum range likely to be encountered in experiments with central fixation.^2^ This value is at least in the range of those reported for CRTs (Ghodrati et al., 2015; their Fig. 3B), better than that obtained for the tested IPS panel (LCD-1; decrease to 80.5%) and significantly better than those reported in older tests of research-grade LCDs (Ghodrati et al., 2015; their Fig. 3B). Finally, we found that just like CRTs and LCDs (e.g., Brainard et al., 2002; Fox et al., 2014; Poth & Horstmann, 2017), the monitor needed to warm up for at least half an hour (e.g., 36 min to reach 99% of its maximum luminance) before any experiment.

### Controlling OLED-specific luminance artifacts

One major complication for precise experimentation with OLED panels is their inherent ABL feature, which regulates the panel’s brightness to balance power usage. Importantly, our measurements suggest that ABL can be avoided by operating the monitor at a moderate brightness setting of 40%, which maps to a luminance of about 140 cd/m^2^. Given that many experiments are conducted in dimmed environments and that the monitor has excellent contrasts, this luminance may be sufficient for many use cases. As an alternative, we found that the monitor’s uniform brightness mode effectively prevented luminance swings due to ABL, allowing users to safely operate the monitor at a luminance of around 260 cd/m^2^. In our tests, this mode only worked reliably under Windows, and not with an otherwise identical Linux setup. However, since similar issues with Linux were not observed in parallel work by Haila and colleagues (Haila et al., 2025), this may have been due to our system configuration. Thus, overall, our results suggest that ABL-like saturation phenomena, already observed in early tests of OLEDs (Cooper et al., 2013; Elze et al., 2013; Ito et al., 2013), can be effectively prevented.

This was not true for another unexpected dimming phenomenon, which we called dynamic dimming. During the first 500 ms of their presentation, bright stimuli decreased in luminance by up to 5%. Interestingly, this decrease was seen exclusively for smaller stimuli subtending 10% or less of the screen. In contrast to ABL, this dimming occurred regardless of the monitor’s brightness setting and regardless of the uniform brightness mode. Our findings on dynamic dimming also match well with independent results obtained by Thauvette and colleagues (2024; see their “SDR brightness” test) as part of a consumer hardware review of the same OLED. Like us, these authors found that small stimuli (2% area) but not large stimuli (≥ 25% area) lose up to 5% of their luminance within the first few seconds. As in our tests, this behavior occurred regardless of the uniform brightness mode. We are unaware of the technical mechanisms causing this seemingly unavoidable dimming of smaller stimuli. However, as mentioned, the magnitude of this luminance change was relatively small (about 5% for the smallest tested stimuli), which is less than, for example, the within-cycle luminance fluctuations observed for the LCD models tested here (see Fig. 1B). It is therefore likely tolerable for many experimental applications.

### Localized warming creates luminance artifacts on longer time scales

A novel and unexpected finding of the present study was a slow, temperature-dependent drift in luminance that emerged when the OLED displayed bright or dark stimuli for extended periods (tens of seconds to minutes). We found that when large bright regions are shown for long durations, the local panel temperature increases, leading to a gradual rise in luminance; conversely, prolonged dark regions cool down and dim. Because this heating or cooling is spatially localized, previously bright areas remain slightly brighter (and previously dark areas slightly darker) even when a subsequent stimulus is shown. The result is a subtle and spatially blurry but systematic form of transient image persistence, mediated by thermal mechanisms. In our measurements, these artifacts were small in absolute magnitude (about 2–3 cd/m^2^, corresponding to roughly 1% of peak luminance), but they decayed only slowly, over tens of seconds. This technical phenomenon has been described at least once in the electrical engineering literature (Chesterman et al., 2016), but to our knowledge, it has not previously been considered in any tests of monitors for visual stimulation.

While likely imperceptible under most conditions, these temperature–luminance interactions could be problematic for certain experiments that rely on long stimulus exposures and very precise control of luminance, such as adaptation paradigms or threshold measurements. Because the effect arises from the panel’s electrothermal characteristics, specifically, temperature-dependent changes in the thin-film transistor current and OLED efficiency (Chesterman et al., 2016), it may affect most or all modern OLED displays, not just the model tested here.

More research is needed to quantify these slow luminance–temperature interactions across different OLED monitors and operating conditions and to determine how artifacts might be mitigated (e.g., by cooling). Our current tests were conducted at a maximum brightness setting of 100%. One simple option to reduce these artifacts may be to operate the monitor at a lower brightness setting, so that lumnious  regions warm up less. For now, researchers using OLEDs for visual stimulation should be aware of this subtle source of luminance drift and consider it when designing experiments that require very stable and spatially uniform output over long periods.

### Temporal properties support fast, time-critical paradigms

Finally, we also ran two practical tests to assess the monitor’s performance in time-critical paradigms. In a first test, we implemented a high-frequency flicker stimulation resembling that used in rapid invisible frequency tagging (RIFT). Originally, RIFT was implemented with a 1,440 Hz projector which can show a 60 Hz sinusoidal flicker sequence at 24 frames/cycle (Minarik et al., 2023; Seijdel et al., 2023). Recently, it was demonstrated that attention-modulated RIFT signals can also be measured in EEG (Arora et al., 2024) at refresh rates of 480 Hz, yielding eight frames per cycle. The tested 240 Hz OLED can already present 60 Hz flicker at four frames per cycle, allowing for the inclusion of two intermediate gray states per cycle to reduce visibility. Due to its short transition times, the OLED was able to properly display each luminance state for most of the 4.17-ms frame duration, a feat likely unachievable with an LCD. While a 240 Hz refresh rate is still insufficient for simultaneous attentional tracking at multiple neighboring frequencies (e.g., at 60 and 64 Hz; Arora et al., 2024), this issue can be addressed by even faster 480 Hz OLED models that have recently been released (Dimigen et al., 2026). More generally, the RIFT test shows that the monitor excels in fast-paced paradigms.

Our second practical test was a gaze-contingent eye-tracking experiment that required the low-latency presentation of a stimulus during a saccade. In our study, the time needed to detect that the eye was moving (*SaccOn* to *SaccDetect* interval) was 5.28 ms. This interval is independent of the monitor used, and its exact duration also depends on the thresholds chosen to detect saccades in the offline analysis. Nevertheless, the low value of around 5 ms confirms the good performance of the online algorithm proposed by Schweitzer and Rolfs (2020) and suggests that this method is an alternative to classic “boundary techniques” (Rayner, 1975) where the saccade is only detected once the gaze exceeds a certain pixel limit.

More important in the present context was the display change latency, which depends critically on the monitor’s refresh rate, input lag, and rise time. Once the saccade was detected, the monitor was able to show the new stimulus at > 90% luminance within 7.61 ms. For comparison, a monitor like the LCD-1, with its 144 Hz refresh rate, would have delayed this change by at least another 12 ms.^3^ The value of 7.61 ms is significantly better than what we have previously achieved in our research with fast CRT monitors (e.g., 9.7 ms in Dimigen et al., 2012; 10.7 ms in Kornrumpf et al., 2016)^4^. It is also shorter than the latency reached with a high-speed projector by Schweitzer and Rolfs (2020)^5^.

Summing up the two intervals, the total latency from saccade onset to stimulus onset was below 13 ms. In practical terms, this meant that we could present the stimulus for the majority of the saccade duration, but not before or after, and also that fewer trials were lost due to late changes.

In principle, these good timing properties should also allow researchers to present multiple stimuli during a saccade, which could alleviate some methodological concerns about saccade-contingent paradigms. For example, to measure the preview benefit from parafoveal information, eye movement studies on reading often compare a condition with a correct pre-saccadic preview to those with an invalid preview. Because only the invalid conditions involve a change in the display, it is at least conceivable that this additional intra-saccadic transient contributes to the preview effects seen in fixation times (e.g., via oculomotor inhibition; Inhoff et al., 1998; O’Regan, 1990; Reingold & Stampe, 2000; Vitu-Thibault, 2007) or neural responses (Chase & Kalil, 1972; Dimigen et al., 2012; Michael & Stark, 1967). The tested OLED would be fast enough to insert an additional intra-saccadic transient (e.g., 1–2 frames of an x-letter mask) in *all* preview conditions (Buonocore et al., 2020), thereby matching them better in terms of low-level stimulation.

### Not tested: Motion, color, and longevity

Our tests focused on timing properties for monochromatic stimuli; motion and color were not examined. To reduce motion blur, it is generally advantageous to use a strobing display with a duty cycle that lasts only a fraction of the refresh cycle. This was the case for the older 60 Hz OLEDs tested for vision research (Elze et al., 2013; Matsumoto et al., 2014). In contrast, the model tested here is a “sample-and-hold” display, meaning that each image is displayed for the full frame duration (see Fig. 1A). In many ways, this is a desirable property for a research display, since the stimulus durations actually match the nominal duration specified by the experimenter (Elze, 2010a). Although sample-and-hold displays result in more motion blur than strobing displays, the high refresh rate and fast transition times of the tested panel should largely mitigate this issue. Tests of color reproduction for the same OLED were conducted by Haila et al. (2025), who report a wide color spectrum and good color accuracy.

Finally, we were not able to test the panel’s longevity. As mentioned, OLEDs are more vulnerable to thermal and electrical stress than other types of panels, and degradation of their organic materials may cause long-term issues such as burn-in or color shifts (Kam et al., 2023; Laaperi, 2008). While several screen protection features must be disabled for precise stimulation, others, such as “pixel cleaning,” can be run between experiments. Whether or not there is a serious risk of burn-in with typical experimental usage at moderate brightness settings is unclear. In any case, it seems prudent to occasionally verify and recalibrate the monitor’s output.

### Conclusions

In our test, the latest generation of consumer-grade high-speed OLED gaming monitors demonstrated excellent temporal properties with short input lags and CRT-like transition times for grayscale stimuli. The self-lit pixels of OLEDs support high contrasts and provide good viewing angles and luminance uniformity. With its 240 Hz refresh rate, the tested monitor seems suitable for paradigms in cognitive science requiring the fast presentation of stimuli with finely graded durations, as demonstrated here for a saccade-contingent paradigm. To prevent luminance artifacts, the monitor needs to be run within a special uniform brightness mode or, alternatively, at moderate brightness settings. We also identified temperature-mediated luminance artifacts that may be problematic for specific experiments which involve long exposures to high-contrast stimuli.

Notably, our findings are broadly consistent with those reported in parallel work by Haila et al. (2025), who independently characterized OLED displays for vision research and also tested the same monitor. The two studies are complementary: for instance, as noted above, Haila and colleagues provide a detailed assessment of color accuracy and calibration, whereas the present work focuses more on temporal aspects of display performance.

Finally, it is significant that modern consumer OLEDs cost only a fraction of dedicated LCDs and projector systems developed for vision science and EEG recordings. The strong performance of OLED technology in fast stimulation paradigms can help to level the playing field by enabling researchers to implement time-critical paradigms regardless of their budget. We also hope that the present battery of tests will serve as a useful template for the evaluation of future OLED displays.

## Data Availability

Data and MATLAB code to reproduce all results and figures are available at https://osf.io/8h4fg/

## Footnotes

1 The resolution of possible stimulus durations could be even further enhanced by using the monitor together with the variable refresh rate feature of modern graphics cards, as proposed by Poth et al. (2018). Because this approach requires that the stimulus lasts at least one frame and that its onset is predictable, it is not feasible in tasks where the stimulation is triggered by behavior, as in our saccade task.

2 At a 50 cm viewing distance, the left and right edges of the 27-inch OLED screen are located at ± 30.5°.

3 This is the sum of a cycle duration (flip latency) that is 2.78 ms longer at 144 Hz plus a much longer flip-to-90% duration of 9.25 ms (see Fig. 1B).

4 Some authors in reading research have reported shorter latencies with much slower monitors. We suspect that these measurements did not consider the time that the cathode ray needs to travel back to the vertical screen center, where sentences were presented.

5 State-of-the-art projectors can display preprogrammed stimulus sequences at 1,440 Hz, but they can only react to real-time user behavior at the slower rate of 120 Hz in this mode (Schweitzer & Rolfs, 2020).

## Code availability

Code is available; see statement regarding availability of data and materials.

## Declarations

### Open practices statement

Data and MATLAB code to reproduce all results and figures are available at https://osf.io/8h4fg/
